# Efficient inhibition of murine breast cancer growth and metastasis by gene transferred mouse survivin Thr34→Ala mutant

**DOI:** 10.1186/1756-9966-27-46

**Published:** 2008-09-25

**Authors:** Xing-Chen Peng, Li Yang, Li-Ping Yang, Yong-Qiu Mao, Han-Shuo Yang, Ji-Yan Liu, Dong-Mei Zhang, li-Juan Chen, Yu-Quan Wei

**Affiliations:** 1 State Key Laboratory of Biotherapy and Cancer Center, West China Hospital, West China Medical School, Sichuan University, Keyuan Road 4, Chengdu, Sichuan, PR China

## Abstract

**Background:**

Metastasis in breast cancer is a vital concern in treatment because most women with primary breast cancer have micrometastases to distant sites at diagnosis. As a member of the inhibitor of apoptosis protein (IAP) family, survivin has been proposed as an attractive target for new anticancer interventions. In this study, we investigated the role of the plasmid encoding the phosphorylation-defective mouse survivin threonine 34→alanine mutant (Msurvivin T34A plasmid) in suppressing both murine primary breast carcinomas and pulmonary metastases.

**Methods:**

In vitro study, induction of apoptosis by Msurvivin T34A plasmid complexed with cationic liposome (DOTAP/Chol) was examined by PI staining fluorescence microscopy and flow cytometric analysis. The anti-tumor and anti-metastases activity of Msurvivin T34A plasmid complexed with cationic liposome (DOTAP/Chol) was evaluated in female BALB/c mice bearing 4T1 s.c. tumors. Mice were treated twice weekly with i.v. administration of Msurvivin T34A plasmid complexed with cationic liposome (DOTAP/Chol), PORF-9 null plasmid complexed with cationic liposome (DOTAP/Chol), 0.9% NaCl solution for 4 weeks. Tumor volume was observed. After sacrificed, tumor net weight was measured and Lung metastatic nodules of each group were counted. Assessment of apoptotic cells by TUNEL assay was conducted in tumor tissue. Microvessel density within tumor tissue was determined by CD31 immunohistochemistry. Alginate-encapsulated tumor cells test was conducted to evaluate the effect on angiogenesis. By experiment of cytotoxicity T lymphocytes, we test whether Msurvivin T34A plasmid complexed with cationic liposome (DOTAP/Chol) can induce specific cell immune response.

**Results:**

Administration of Msurvivin T34A plasmid complexed with cationic liposome (DOTAP/Chol) resulted in significant inhibition in the growth and metastases of 4T1 tumor model. These anti-tumor and anti-metastases responses were associated with triggering the apoptosis of tumor cells directly, inhibiting angiogenesis and inducing specific cellular immune response.

**Conclusion:**

The present findings suggest that the Msurvivin T34A plasmid complexed with cationic liposome may provide an effective approach to inhibit the growth and metastases of a highly metastatic mouse breast cancer model with minimal side effects.

## Background

Epidemiologic studies indicate 180,510 new cases of breast cancer will be diagnosed and 40,910 will die of breast carcinoma only in the united states in 2007. Recently breast carcinoma has been the most common malignancy in women and is second only to lung cancer as a cause of cancer death[1]. Although breast cancer mortality appears to be declining due to a benefit from early detection and more effective treatment, metastasis after surgical resection plays an important role in the failure of therapy and the death of patients. In view of these, more effective strategies need to be developed. Breast tumours mainly metastasize to the lungs and bones. A murine mammary carcinoma cell 4T1 has a highly metastatic characteristic at an early stage, which is similar to the human breast cancer and make it an excellent animal model to evaluate the effects of drugs on breast tumor metastases as well as growth [2,3].

As a member of the inhibitory apoptotic protein family, survivin is a 16.5 kda antiapoptotic protein. By targeting caspase 9 via interaction with cofactor molecules, survivin tends to hinder mitochondrial-dependent apoptosis[4]. Because of its role in regulation of the balance between programmed cell death and cell proliferation during cell cycle, survivin is vital for cancer cell survival, which makes it an attractive target for new anticancer interventions [4-6]. The protein is strongly expressed in the most common human neoplasms, embryonic and fetal organs but is generally undetectable in differentiated normal tissues with the exception of thymus, basal colonic epithelium and endothelial cells [7-11]. Moreover, the level of survivin protein appears to be involved in tumour cell resistance to ionizing radiation[12] and some anticancer agents[13,14]. Meanwhile, previous studies have demonstrated that the overexpression of survivin correlates with poor survival of patients and poor prognosis[15].

Recently, considerable efforts including antisense[16], ribozymes[17], RNAi-mediated[18,19] survivin knock-down, survivin-directed vaccines[20] or dominant negative mutants[16,21] have been made to counteract survivin in tumor cells in order to inhabit tumor growth potential and enhance tumor cell response to apoptosis-inducing anti-cancer agents. Results from studies exploiting different strategies to interfere with survivin expression and function provided direct and convincing evidence that targeting survivin is a promising approach for cancer therapy. It was suggested that dominant negative mutants including survivin Thr34→Ala, survivin Cys84→Ala could reduce tumor cell proliferative potential and lead to caspase-dependent apoptosis in melanoma cell lines[16,21]. The results of several previous basic studies and phase I trials based on the administration of survivin-directed Cytotoxic T lymphocytes have been completed and proved to be an effective and safe method to move on to clinical phase 2 studies[20,22]. However, no findings that Msurvivin T34A mutant can deal with highly metastatic breast carcinoma have been reported. Compared with intratumor injection reported before, intravenous injection we used can control micrometastases throughout the body and can be extended to the clinical application better. In this study, we focus on this main under-explored question: to test whether Msurvivin T34A plasmid complexed with cationic liposome (DOTAP/CHOL) may provide good anti-tumor effect to murine metastatic mammary carcinoma and inhibit experimental tumor metastases by injection through tail vein.

## Methods

### Cell and cell cultures

The 4T1 mouse mammary carcinoma cells and the murine endothelial cells(MS1) (obtained from American Type Culture Collection, ATCC, Rockville MD) were cultured in Dulbecco's modified Eagle medium (DMEM) (Gibco BRL, Grand Island, N.Y.) supplemented with 10% heat-inactivated fetal bovine serum (FBS), 100 μg/ml amikacin and were maintained in humidified chamber at 37°C in 5% CO_2 _atmosphere.

### Expression vector

The recombinant plasmids carrying PORF-9-Msurvivin T34A or PORF-9 null were purchased from Invivogen Corporation. The recombinant plasmid was confirmed by restriction endonuclease analysis, PCR and DNA sequence analysis (data not shown). The plasmid PORF-9 null without mutant survivin gene was used as a control in the experiment.

### The plasmid DNA preparation

The plasmid was prepared using Endofree Plasmid Giga kit (Qiagen, Chatsworth, CA). Endotoxin levels of the plasmid DNA prepared were determined by Tachypleus Amebocyte Lysate (TAL). No genomic DNA, small DNA fragments, or RNA were detected in the DNA prepared and the OD_260/280 _ratios of the plasmid DNA prepared were between 1.8–2.0. The DNA was eventually dissolved in sterile endotoxin free water and stored at -20°C before use.

### Liposome preparation

DOTAP:chol liposome was prepared using the procedure described previously [23]. Briefly, the cationic lipid DOTAP was mixed with the neutral lipid Chol at equimolar concentrations. The mixed lipids were dissolved in chloroform in a 100 ml-round-bottomed flask. Then, the clear solution was rotated on a Buchi rotary evaporator at 30°C for 30 min to make a thin film, the flask containing the thin lipid film was dried under vacuum for 15 min. The film was hydrated in 5% dextrose in water (D5W) to give a final concentration of 7 mM DOTAP and 7 mM chol, referred to as 7 mM DOTAP:chol. The hydrated lipid film was rotated in a water bath at 50°C for 45 min and then 35°C for 10 min. The mixture was allowed to stand in the parafilm-covered flask at room temperature overnight, after which the mixture was sonicated at low frequency for 5 min at 50°C, transferred to a tube, and heated for 10 min at 50°C. The mixture was sequentially extruded through Millipore (Billerica, MA) polycarbonate membrane of decreasing size: 0.2 μm for 5 times and 0.1 μm for 3 times using syringes. Liposome were stored under argon gas at 4°C. DOTAP was purchased from Avanti Polar Lipids (Alabaster, AL); and highly purified Chol was purchased from Sigma (St. Louis, MO).

### Apoptotic detection *in vitro*

#### Cellular transfection

The recombinant pcDNA3.1 plasmid encoding for the green fluorescent protein was used to find the ideal plasmid:liposome ratio (μg/μg) for the most efficient gene delivery. A maximum expression was obtained when 2μg plasmid/6μg liposome was used (data not shown).

For transfection, 2 × 10^5 ^cells (including 4T1 cells or MS1 cells) were plated into 6-well plates (10 cm^2 ^growth area) and incubated overnight in DMEM to 60% to 70% confluence.2 μg DNA (Msurvivin T34A plasmid or PORF-9 null plasmid) with 6 μL liposome was dissolved in 500 μL of serum-free DMEM without antibiotics for 30 minutes at 25°C and added directly to cell cultures. Cells were then incubated for 6 hours at 37°C, after which, the media were replaced with fresh serum-supplemented media with antibiotics.48 hours later, cells were harvested to be used to apoptotic analysis as described below.

#### Morphological analysis

After transfection, cells were resuspended in hypotonic propidium iodide solution containing 50 micro g PI/ml in 0.1% sodium citrate plus 0.1% Triton X-100 and examined by fluorescence microscopy.

#### The quantitative assessment of apoptosis

Harvested cells were stained with 1 ml hypotonic fluorochrome solution containing 50 micro g/ml propidium iodide in 0.1% sodium citrate plus 0.1% Triton X-100. Then flow cytometric analysis could be performed to identify apoptotic cells or sub-G1 cells and to measure the percentage of sub-G1 cells by the use of a flow cytometer (ESP Elite; Coulter). Apoptotic cells has a less DNA content than that of G1 cells in the cell cycle distribution and the results was estimated with list mode software.

### Animal experiments

Female *BALB/c *mice and *KM *mice of 6 to 8 weeks old were purchased from experimental animal center of Sichuan University (Chengdu, Sichuan province, China) and were housed in our animal research facility. Mice were kept in groups of five per cage and fed with clean food and water. The animals were acclimatized for 1 week before use and maintained throughout at standard conditions: 24 ± 2°C temperature, 50 ± 10% relative humidity.

Female *BALB/C *mice were challenged s.c with 1 × 10^5 ^4T1 mouse breast tumor cells in the right flank on day 0. Primary tumors were palpable on day 7–9 and had a mean diameter of 3 mm on day 9. The tumor-bearing mice were randomly assigned into the following three groups and received the corresponding treatment: (a) mice received 25 μg Msurvivin T34A plasmid/75 μg liposome complexes (volume = 200 μl), (b) mice received 25 μg PORF-9 null plasmid/75 μg liposome complexes (volume = 200μl), and (c) mice received 200 μl 0.9% NaCl solution (NS). Each group has 10 mice. Half an hour before tail vein injection, DNA:liposome complexes were prepared.25 μg DNA was diluted in D5W (dextrose 5% in water), and 75 μg liposome were diluted in D5W to produce 1:3 ratio of DNA (μg): liposome (μg). Equal volumes of both the DNA solution and the liposome solution were mixed gently and incubated at room temperature for 30 min. The systemic therapy started on day 10 and was repeated twice a week for the total of 8 times. The mice were bled via the tail vein at weekly intervals to collect serum. On day 38, all mice from each group were sacrificed. Tumor net weight of each mouse was measured. Tumor growth was evaluated by measurement of tumor diameters every 3 or 4 days and the tumor volume was calculated as length × width^2 ^× 0.52. All of the data are represented as means ± SE.

Autopsy was performed to determine the number and diameter of the metastatic nodules of lung. Organs (lung, liver, kidney, heart and spleen) and tumors from sacrificed mice were removed, fixed in the 4% formaldehyde solution for histologic analysis.

### Histologic analysis

Primary tumors were fixed in 4% paraformaldehyde in PBS, embedded in paraffin and cut into 3–5 μm sections. Then the sections were stained with hematoxylin and eosin (H&E). The presence of apoptotic cells within the tumor sections was evaluated by the TUNEL (terminal deoxynucleotidyltransferase-mediated dUTP nick-end labeling) technique using the DeadEnd Fluorometric TUNEL System (Promega, Madison, WI) following the manufacturer's protocol. Percent apoptosis was determined by counting the number of apoptotic cells and dividing by the total number of cells in the field (5 high power fields/slide).

Immunohistochemistry analyses of microvessel expression were done with goat anti-mouse CD31 antibody (Santa Cruz Biotechnology) using the labeled streptavidin-biotin method. That is to say, Sections were deparaffinized in xylol and rehydrated in graded alcohol series. Antigen retrieval was carried out by autoclaving sections in retrieval buffer (10 mM pH 6.0 EDTA citrate buffer) for 3 min in saturated steam after up-pressure gaining (126°C, 1.6 bars, 23 psi). Endogenous peroxidase activity was blocked by incubation in 3% hydrogen peroxide at room temperature free of light for 20 min. Nonspecific binding of reagents was quenched by incubation of sections for 20 min in 5% normal rabbit serum. Sections were then incubated with goat anti-mouse CD31 (dilution 1/200) antibodies overnight at 4°C, followed by incubating with biotinylated rabbit anti-goat IgG and then streptavidin-biotin-horseradish peroxidase complex at 37°C for 1 hour severally. Negative control was included with each run by substituting the primary antibody with non-immune rabbit serum. Cellular nuclei were counterstained with ameliorative Gill's hematoxylin.

### CD31 immunohistochemical evaluation

According to the method of Weidner *et al*. [24], the quantification of microvessel density (MVD) was assessed. The sections were firstly screened at low magnifications (×40 and ×100) to identify the most vascular area of the tumor called hot spot. Within the hot spot area, the stained microvessel were counted in a single high-power (×400) field. MVD was expressed as the number of microvessel/field. Any CD31-stained endothelial cells or endothelial cell clusters that were clearly separate from adjacent microvessel, tumor cells, or connective tissue elements were considered a single countable microvessel.

### Alginate-encapsulated tumor cells test

As a specific tumor angiogenesis model *in vivo*, the alginate-encapsulated tumor cells test was conducted in order to investigate whether or not the use of Msurvivin T34A plasmid would significantly reduce the angiogenesis at the alginate implant. All *BALB/c *female mice were injected s.c. with 0.1 ml of alginate beads containing 1 × 10^5 ^4T1 cells into the upper third of the back. The next day, mice were randomly assigned into one of the following three groups (n = 5): (a) mice received 25 μg Msurvivin T34A plasmid/75 μg liposome complexes (volume = 200 μl), (b) mice received 25 μg PORF-9 null plasmid/75 μg liposome complexes (volume = 200 μl), and (c) mice received 200 μl 0.9% NaCl solution (NS). Treatment of Msurvivin T34A plasmid (twice/week) started on the same day. Two weeks later, 200 μl of 1% FITC-dextran solution (100 mg/kg) was injected i.v. into the tail vein of mice. All the alginate implants were removed 20 min after FITC-dextran injection as soon as possible. Image of the alginate implants was taken by using SPOT FIEX camera. Alginate beads were transferred to tubes containing 2 ml of saline. The tubes were mixed by a vortex for 20 s and centrifuged (3 min; 1000 × g). Finally the fluorescence of the supernatant was measured to quantify blood vessel formation.

### Cytotoxic T lymphocytes assay

According to the methods described in [25,26], mononuclear cells of spleen were harvested and depleted of red cells with ammonium chloride and passed through nylon wool after the mice were sacrificed. Effector cells, T cells of spleen, were prepared. Target cells 4T1 were labeled with 300 mci of Na_2 _^51^CrO_4 _for 2 h, washed, and dispensed into the wells of 96-well plates. Different number of the effector cells were added in duplicates to generate different E:T ratios. The radioactivity released into supernatants was measured in a scintillation counter.

### Toxicity assessment

In order to evaluate the potential side effects in the Msurvivin T34A plasmid:lipo complexes-treated mice, they were continuously observed for relevant indexes such as weight loss, ruffled fur, diarrhea, anorexia, skin ulceration and toxic death. After mice were sacrificed, various organs (heart, liver, spleen, lung, kidney, brain, etc.) were harvested and fixed in 4% paraformaldehyde in PBS. These tissues were then sectioned, stained with H&E and observed by two pathologists in a double- blinded manner. In order to evaluate hematologic toxicity, blood test was conducted. In addition, the acute toxicity was also assessed. Some *KM *mice about 6–8 weeks old were randomly assigned to the following three groups and received the corresponding treatment: (a) mice received 25 μg Msurvivin T34A plasmid/75 μg liposome complexes (volume = 200 μl), (b) mice received 25 μg PORF-9 null plasmid/75 μg liposome complexes (volume = 200 μl), and (c) mice received 200 μl of 0.9% NaCl solution (NS). Every group has 6 mice. Blood samples were collected from the retro-orbital vein 48 hours after the injection. The levels of serum ALT and AST were determined with an automatic multifunction-biochemical analyzer.

### Statistical analysis

Statistical analysis of the differences in tumor volume, tumor net weight, animal weight, percent apoptosis, microvessel density and the level of serum transaminase were performed using one-way analysis of variance (ANOVA). Because distribution of metastatic tumors in the lungs is not normal, the data have to be presented as medians, and statistical analysis of the differences in metastasis formation was analyzed using the Mann-Whitney test. A *P *< 0.05 was considered statistically significant.

## Results

### Apoptotic morphological observation

**Figure 1:**
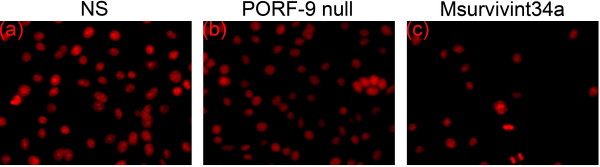
**Fluorescence microscopic appearance of propidium iodide (PI)-stained nuclei**. 4T1 cells were treated with Msurvivin T34A plasmid(2 μg per well)for 48 hr. (a) untransfected (b)transfected with PORF-9 null plasmid(c)transfected with Msurvivin T34A plasmid, reduction of cell volume, condensation of nuclear, nuclear fragmentation, and apoptotic bodies.

Treatment with Msurvivin T34A plasmid of tumor cells 4T1 contributed to morphological changes characteristic for apoptosis: brightly red-fluorescent nuclei (fragmented or intact) displayed by fluorescence microscopy of PI-stained nuclei, reduction of cell volume, condensation of nuclear chromatin, nuclear fragmentation and apoptotic bodies. By contrast, the other groups (including the untransfected and the transfected with PORF-9 null plasmid) did not demonstrate obvious changes (Figure 1).

### Flow cytometry analysis

**Figure 2:**
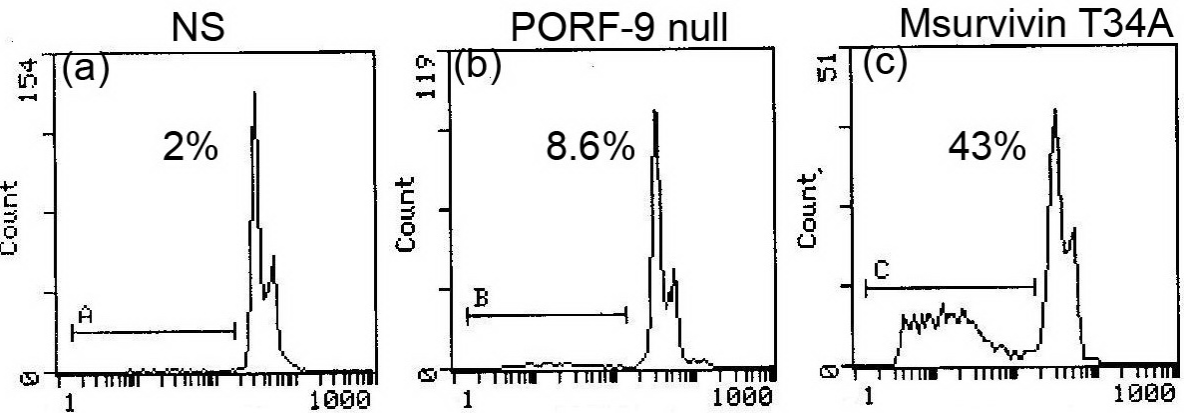
**Representative DNA fluorescence histograms of PI-stained cells**. 4T1 cells were treated with Msurvivin T34A plasmid(2 μg per well)for 48 hr.(a)untreated,(b)treated with PORF-9 null plasmid,(c)treated with Msurvivin T34A plasmid, with(a) 2%,(b)8.6% and(d) 43% sub-G1 cells(apoptotic cells), respectively, as assessed by flow cytometry.

The quantitative assessment of sub-G1 cells by flow cytometry was used to estimate the number of apoptotic cells.4T1 cells treated with Msurvivin T34A plasmid for 48 hr. have a striking sub-G1 peak compared with the other groups (including the untransfected and the transfected with PORF-9 null plasmid), with 43%, 2% and 8.6% sub-G1 cells (apoptotic cells), respectively, as assessed by flow cytometry (Figure 2).

### Anti-tumor effect of Msurvivin T34A plasmid *in vivo*

**Figure 3:**
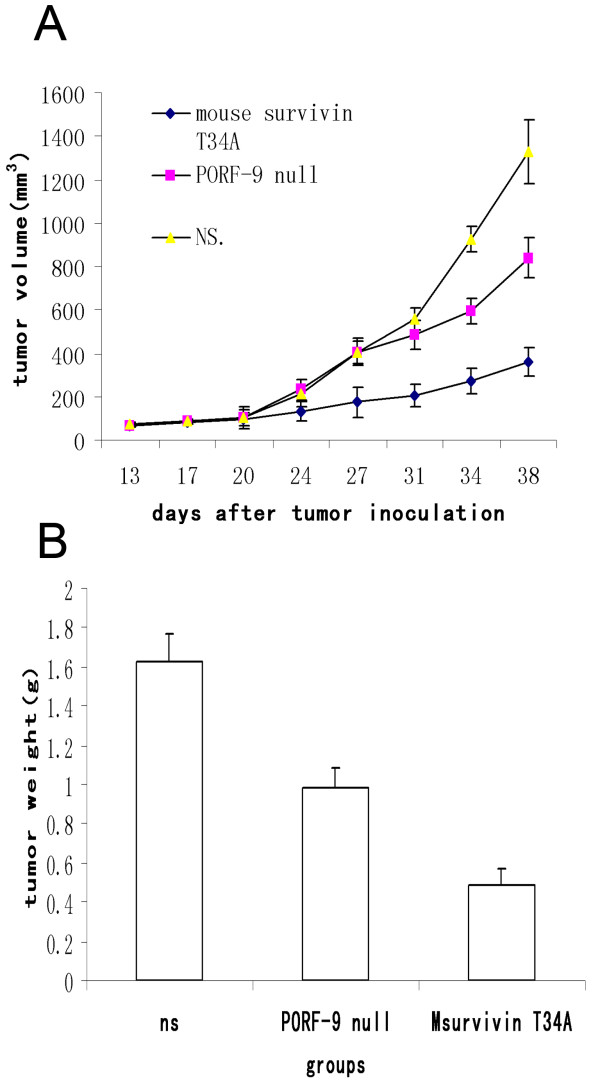
**Anti-tumor effects of Msurvivin T34A plasmid:lipo complexes versus normal saline, PORF-9 null plasmid:lipo complexes**. The tumor-bearing mice in each group received the corresponding treatment as detailed in "Materials and Methods". Data were presented as means ± SE. Each group contained 10 mice.(A) Tumor volume was shown up to 38. The differences between Msurvivin T34A plasmid:lipo and control groups were significant (*P *< 0.05) starting day 27.(B)Tumor weight was measured after each mice from every group was sacrificed. Compared with control groups, the Msurvivin T34A plasmid treated mice showed significant difference. (P < 0.01)

In order to examine the applicability of Msurvivin T34A plasmid complexed with cationic liposome(DOTAP/Chol)*in vivo *system, we established the murine metastatic breast tumor model by subcutaneous inoculation of 1 × 10^5 ^tumor cells.10 days later, mice were randomized to receive administration of Msurvivin T34A plasmid, PORF-9 null plasmid and normal saline separately. Tumor volume presented in were monitored every 3 to 4 days (Figure 3A). At the termination of the experiment, all the animals were sacrificed and the tumor weight of each mouse from the three groups were measured (Figure 3B). It is obvious that the treatment with Msurvivin T34A plasmid resulted in primary tumor growth regression of 73% and 57%, respectively, compared with NS and PORF-9 null plasmid groups(P < 0.05). The corresponding results were also found in the tumor weight. We observed the average weight of the tumors in Msurvivin T34A plasmid treated group was reduced by 68% and 46% when compared with the NS and PORF-9 null plasmid groups(P < 0.01). All the data showed that the Msurvivin T34A plasmid has a significant influence on the supression of tumor growth.

### Msurvivin T34A plasmid inhibits the metastasis of 4T1 fromthe primary tumor site to the lungs *in vivo*

**Figure 4:**
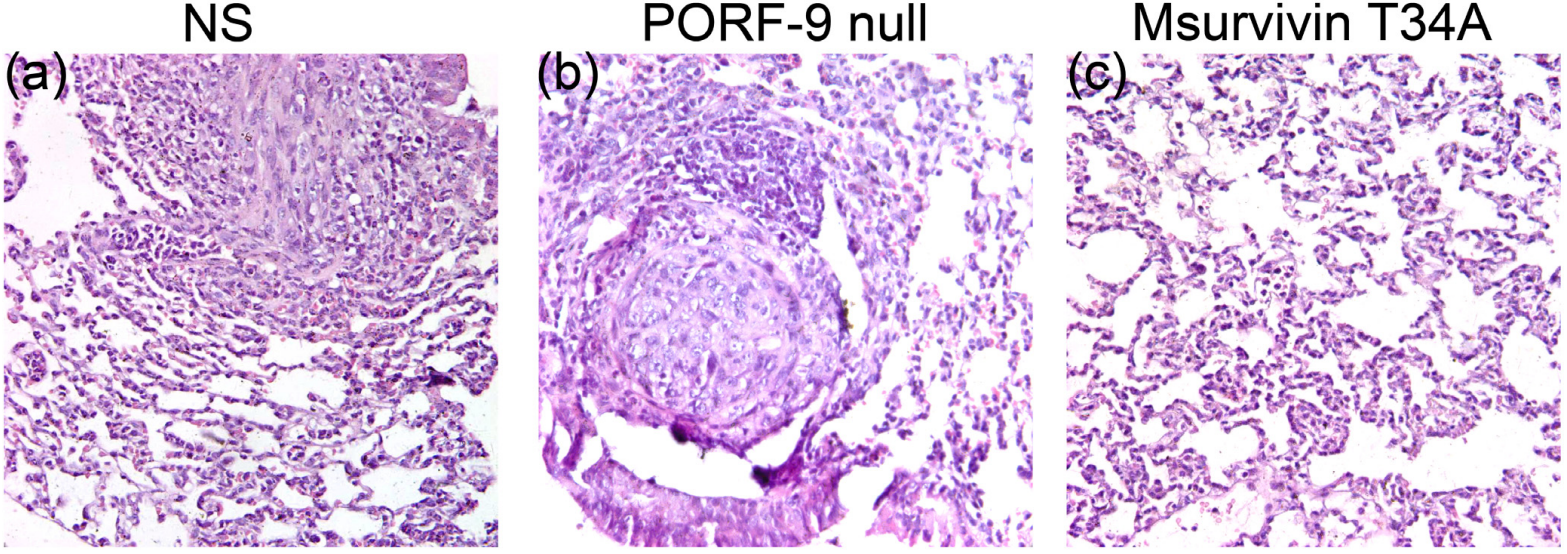
**The anti-metastases effect of Msurvivin T34A plasmid:lipo complexes**. (a) NS group, (b) PORF-9 null plasmid group, (c) Msurvivin T34A plasmid group. Compared with Msurvivin T34A plasmid group, a large number of lung metastatic nodules and structural destruction of pulmonary alveoli can be observed in NS and PORF-9 null plasmid group.

Since the 4T1 breast tumor cells have been confirmed to have the large potential to metastasize to the lung, liver, brain and so on within 2 weeks [27-29], the therapeutic efficacy against metastases was evaluated at the day 38, when mice were sacrificed and lungs, livers, hearts, spleens and kidneys were carefully examined. Moreover, the tumor nodules on the lungs were counted, for some studies have showed the metastases to the lungs can represent the extent of the cell spreading throughout the mice [3,30]. From the results of examination, we found that the NS group had a median of 37 metastases/lungs(range, 22–45 metastases), and the median of PORF-9 null plasmid group is 34 metastases/lungs(range, 19–46 metastases), whereas mice treated with Msurvivin T34A plasmid had a median of 5 metastases/lungs(range,0–9 metastases), respectively. To our delight, 4 of 10 mice had no visible lung metastases in the group treated with Msurvivin T34A plasmid (Table 1). The results of H&E staining of lungs conformed to the visible observation (Figure 4A). These results indicate that Msurvivin T34A plasmid has a potent ability to stimulate anti-tumor and anti-metastases activity in these mice with established 4T1 breast tumor.

**Table 1:** Lung metastatic nodules of each group

Groups	Median no. of metastases (range)/lung	% metastasis-free animals
Normal saline	35 (22–45)	0
PORF-9 null plasmid:lipo complexes	31.5 (19–46)	0
Msurvivin T34A plasmid:lipo complexes	5 (0–9)*	40*

*significantly (*P *< 0.05) differs from the control groups according to Mann-Whitney test

### Inhibition of tumor-induced angiogenesis (CD31) and increase of apoptosis (TUNEL)

**Figure 5:**
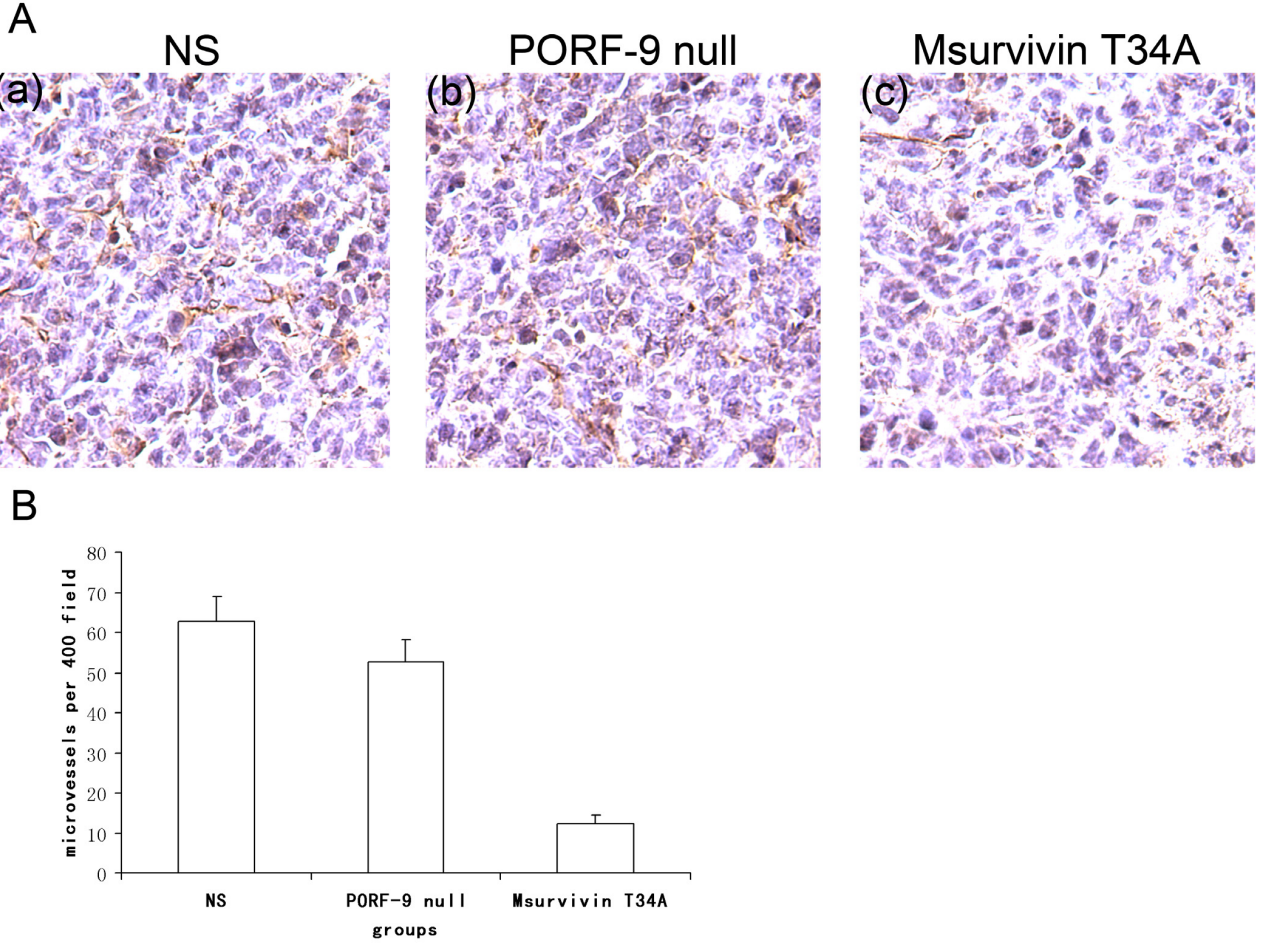
**Inhibition of intratumoral angiogenesis assayed by CD31 staining of microvessel**. Vascularization within tumors was detected by an antibody to CD 31 and vascular density was quantified by counting the number of microvessel per high power field (×400). (A) Shown are representative sections from each group. (a)NS group, (b) PORF-9 null plasmid group, (c) Msurvivin T34A plasmid group. The arrows point to the necrosis. (b) CD31 positive microvessel in each group. Values were expressed as means ± SE (5 high power fields/slide).

**Figure 6:**
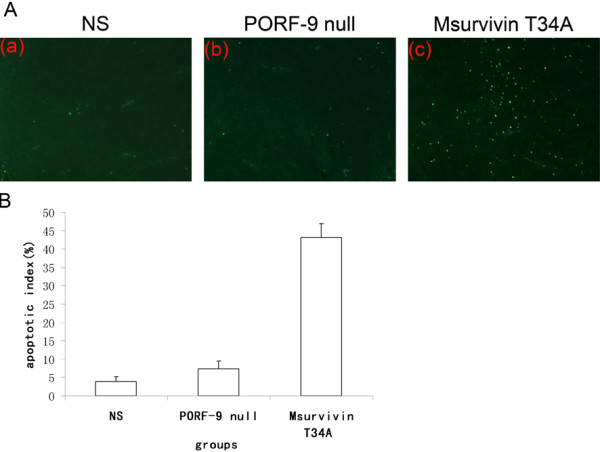
**Terminal deoxynucleotidyltransferase-mediated dUTP nick-end labeling staining of tumor tissues**. Sections after treatment were stained by TUNEL analysis to detect apoptotic cells. Percent apoptosis was determined by counting the number of apoptotic cells and dividing by the total number of cells in the field (5 high power fields/slide). (A) Representative Field from each group. (a) NS group, (b) PORF-9 null plasmid group, (c) Msurvivin T34A plasmid group. (B) Percent apoptosis in each group. Values were expressed as means ± SE. Msurvivin T34A plasmid:lipo complexes increased the percent apoptosis in breast tumor sections relative to either normal saline or PORF-9 null plasmid:lipo complexes. (*p *< 0.05, respectively).

Tumor sections of each group were stained with anti-CD31 antibody (Figure 5A) and TUNEL reagent (Figure 6A) in order to evaluate microvessel density (MVD) and apoptosis rate as described above. Tumor of the control groups(including NS and PORF-9 null plasmid groups) demonstrated high microvessel density while those of the Msurvivin T34A plasmid group had decreased values(P < 0.05) (Figure 5B). Meanwhile we can find a large area of necrosis in the section of Msurvivin T34A plasmid treated group. There was much more apoptotic cells seen in those from Msurvivin T34A plasmid treated group than in the control groups. Furthermore, tumors from animals that received Msurvivin T34A plasmid tended to display the highest apoptotic indices(P < 0.05) (Figure 6B). These data can suggest mouse Msurvivin T34A plasmid can cause the inhibition of angiogenesis and directly increase apoptosis of tumor cells, which play an important role in the treatment of breast carcinoma.

### Apoptosis induction of Msurvivin T34A plasmid on murineendothelial cells (MS1) *in vitro*

**Figure 7:**
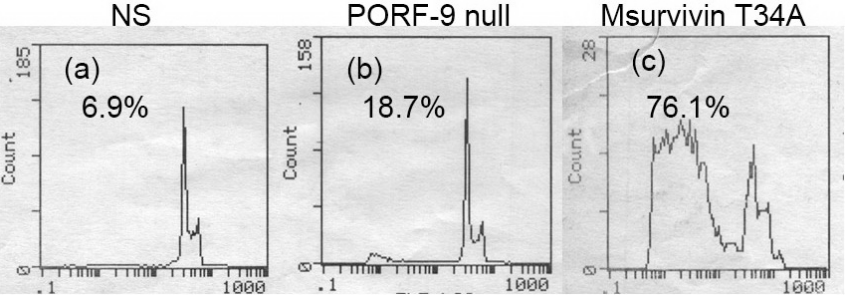
**Msurvivin T34A plasmid induced apoptosis of murine endothelial (MS1) cells *in vitro***. MS1 cells were treated with Msurvivin T34A plasmid(2 μg per well)for 48 hr.(a)untreated,(b)treated with PORF-9 null plasmid,(c)treated with Msurvivin T34A plasmid, with(a) 6.9%,(b) 18.9%and(d) 76.1% sub-G1 cells(apoptotic cells), respectively, as assessed by flow cytometry.

In order to investigate the mechanism by which the Msurvivin T34A plasmid affected angiogenesis, the impact of Msurvivin T34A plasmid on murine endothelial cells (MS1) was tested after the transfection as described above. The characteristic changes such as a brightly red-fluorescent condensed nuclei(intact or fragmented), reduction of cell volume and apoptotic bodies can be observed in the Msurvivin T34A plasmid treated group after the PI-staining(data not shown). The numbers of apoptotic cells were quantitatively estimated by observing sub-G1 cells by the flow cytometry. The results displayed that there were a much lager amount of apoptotic cells in the Msurvivin T34A plasmid treated group than in the others(including the PORF-9 null plasmid and the PBS treated groups)(Figure 7)

### Alginate-encapsulated tumor cells test

**Figure 8:**
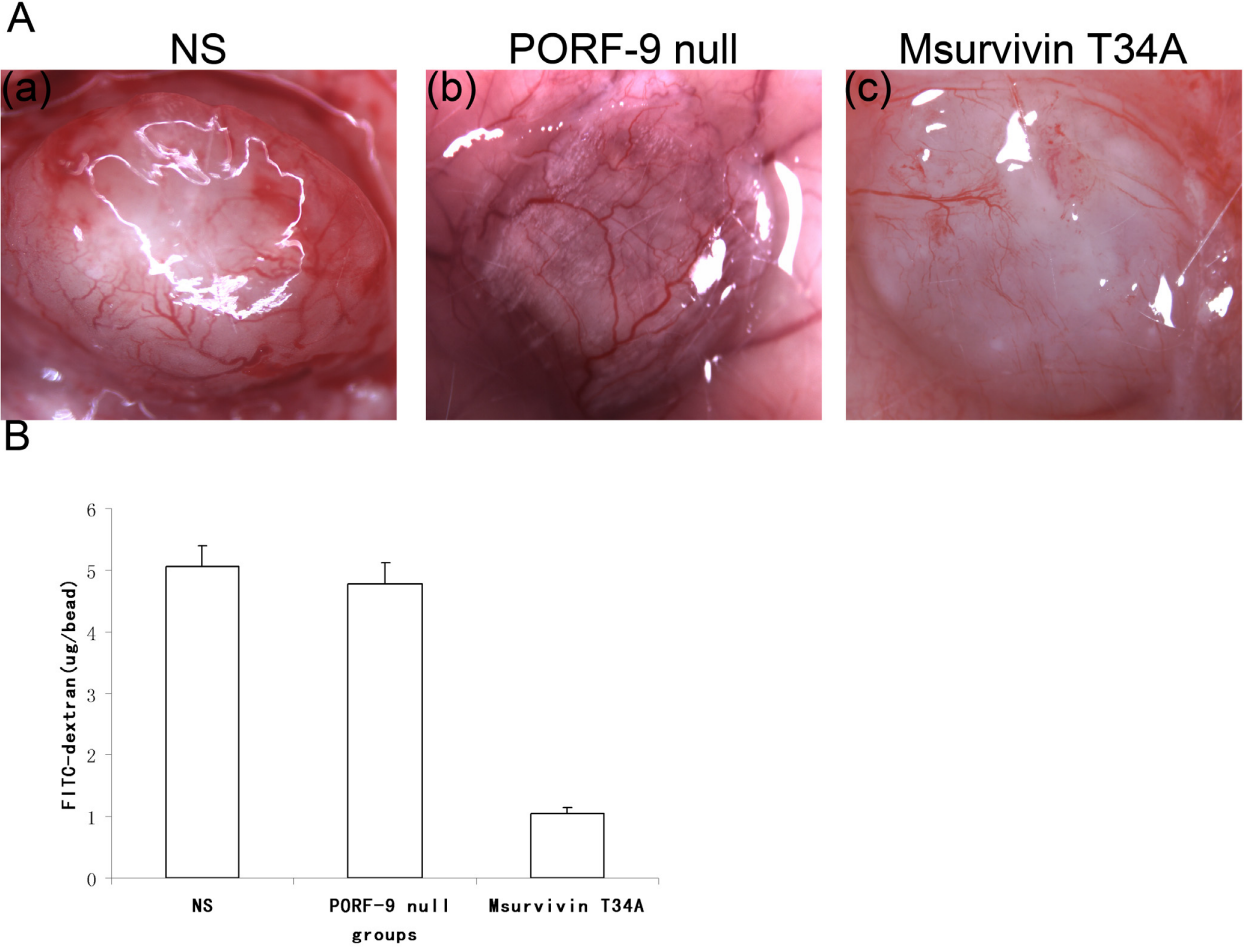
**Vascularization of alginate implants**. (A) Representative alginate beads from each group.(a)NS group,(b)PORF-9 plasmid null plasmid group,(c)Msurvivin T34A plasmid group.(B) FITC-dextran uptake of beads from Msurvivin T34A plasmid treated mice showed a significant decrease compared with control groups(P < 0.05). The data were expressed as means ± S.E.

Alginate can not only provide the tumor cells and the growth of microvessel with ground substance, but also can not affect the growth of tumor cells and the infiltration of cell factor and drugs. The results of vascularization of alginate implants demonstrated that Msurvivin T34A plasmid treated mice had a decrease of new vessels compared with control groups, which conformed to the results of FITC-dextran uptake of beads. (P < 0.05) (Figure 8)

### Experiment of cytotoxicity T lymphocytes

**Figure 9:**
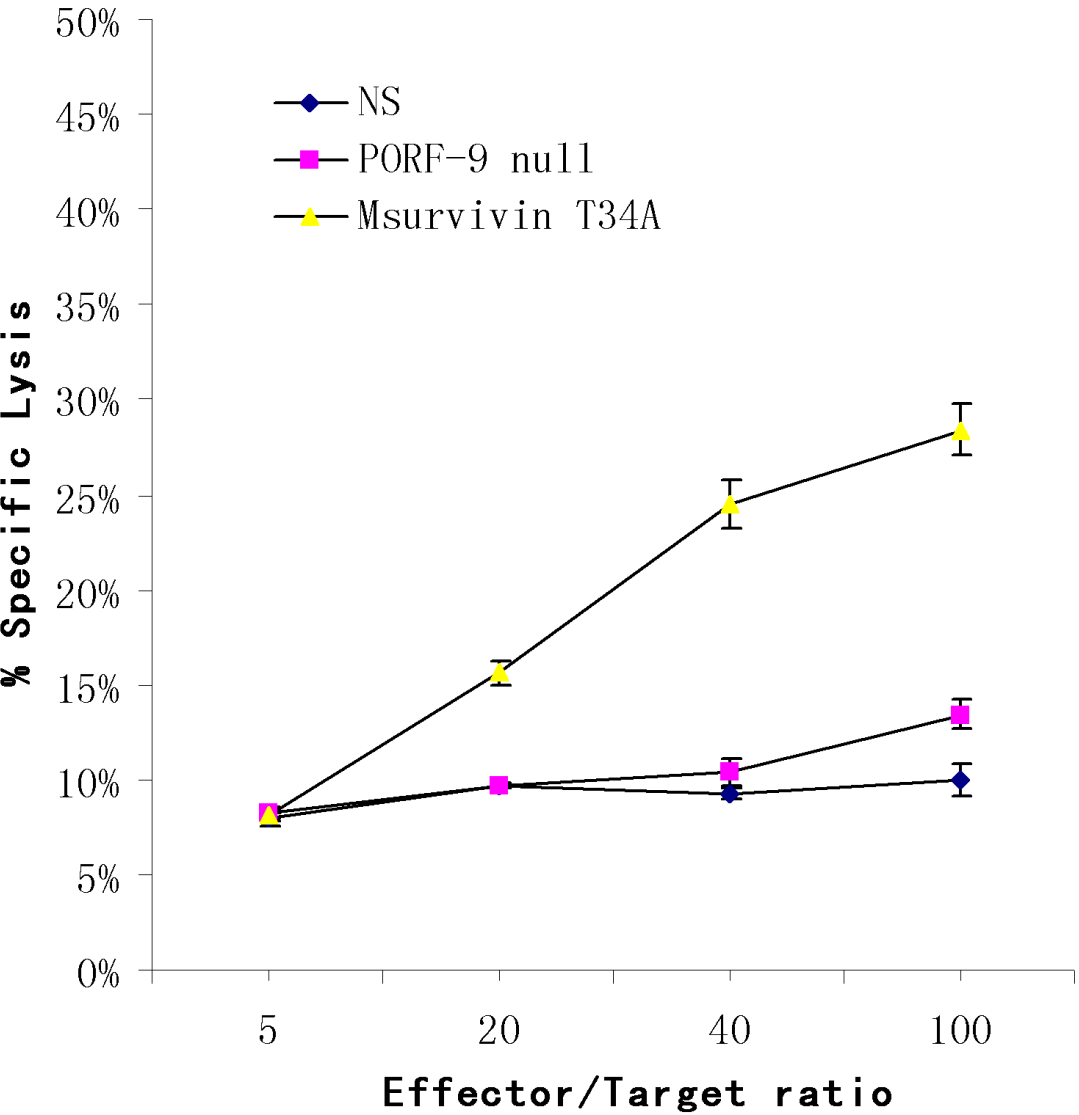
**Msurvivin T34A plasmid induces a specific cellular immune response**. Msurvivin T34A plasmid showed increased cytotoxicity against survivin-positive target cells while the other groups did not induce specific cytotoxicity reaction. (P < 0.05) The data were expressed as means ± S.E. of triplicate samples from one representative experiment.

In order to investigate whether Msurvivin T34A plasmid could induce specific cellular immune response, we made use of Cr^51 ^release assay to examine primed T cells to assess the cytotoxicity activity. Msurvivin T34A plasmid treated group showed higher killing activity against autologous tumor cells, while T cells from the control group failed to show any significant killing activity.(P < 0.05)(Figure 9A)

### Toxicity assays

As the survivin can be expressed in adult normal tissue such as hematopoietic progenitor cells, basal colonic epithelial cells etc. and as a systematic therapic method against tumors, injection through tail vein may lead to the occurrence of systematic toxicity, we attempted to measure the toxicity of the treatment. Blood test did not show obvious differences among three groups. The acute toxic reaction of liver were evaluated by measuring the levels of serum AST and ALT of *KM *mice 48 h after receiving corresponding treatments as described above. NO significant differences between Msurvivin T34A plasmid treated group and the control mice were found. (P > 0.05) (data not shown). Body weights of mice were measured by a digital balance every 3 or 4 days. The weight curve of each group paralleled very closely. (data not shown) No gross abnormalities were observed in the Msurvivin T34A plasmid treated group. Furthermore, no toxic pathologic changes in heart, liver, spleen, lung and kidney were detected by two pathologists among the three groups.

## Discussion

As the most vital attribute of malignant tumor, metastases have been a continuing therapeutic challenge, due to the limited therapeutic effects of the modalities available now. With regard to breast cancer, the most common malignancy affecting women throughout the world, numerous data suggest that when metastases occur, it always means poor prognosis despite further treatment such as surgery, radiotherapy, endocrinotherapy or traditional chemotherapy, though they have some curative effects on breast cancer, especially in its early stages. In a word, there is an imperative need to develop more effective agents to deal with combat metastatic breast cancer better. One such approach is gene therapy.

Among the regulators of apoptosis that may participate in cancer, survivin has attracted increasing attention because of its essential role that is implicated in all kinds of cancer survival, diagnosis and cancer treatment [4-6]. As a suppressor of apoptosis, survivin is strongly expressed in the most common human neoplasms [7-11], has prognostic relevance for most cancers[15]and appears to be involved in the response of tumor cells to chemotherapy and radiation [12-14]. Furthermore, the expression of survivin in normal adult tissues can not be detected or significantly lower than in transformed cells[7]. So it is not difficult to understand why survivin targeted treatment can not lead to systemic toxicity. It has been reported that down-regulation of survivin expression or function could reduce tumor growth potential, increase the apoptotic rate and sensitize tumor to chemotherapy and radiation in different tumor models [16-20]. Mouse survivin T34A is a phosphorylation-defective Thr34→Ala dominant negative mutant. Grossman et al. first demonstrated that transfection of YUSAC2 and LOX melanoma cell lines with a mutant carrying a cysteine 84→alanine mutation in the survivin baculovirus IAP repeat domain increased the apoptotic index and enhanced the sub-G1 apoptotic cell fraction in both tumor models. Later, Grossman et al. demonstrated the induction of spontaneous apoptosis in different melanoma cell lines as well as an increased apoptotic response following cisplatin treatment by using a phosphorylationdefective survivin threonine 34→alanine mutant [21]. The mechanisms by which the Msurvivin T34A induces apoptosis are mitochondrial-dependent apoptosis with release of cytochrome c and loss of mitochondrial transmembrane potential by targeting caspase 9 and other molecules [4]. Here, for the first time, we evaluated the therapeutic efficacy of Msurvivin T34A plasmid:lipo complexes on mouse metastatic breast cancer 4T1 by intravenous injection and found the Msurvivin T34A plasmid had potent anti-tumor and anti-metastases activity against mouse metastatic breast cancer model *in vitro *and *in vivo *by triggering apoptosis of cancer cells, inhibiting angiogenesis and inducing specific cellular immune response without obvious systemic toxicity.

The novel and efficient gene delivery system that we utilized in present study is cationic liposome which increases the therapeutic efficacy of Msurvivin T34A plasmid but does not contribute to obvious systemic toxicity. As a nonviral gene delivery system, Cationic liposome has low immunogenicity, minimal toxicity. It has several advantages. Firstly, among various formulations of cationic liposome, it has been reported that DOTAP:Chol can achieve higher levels of expression in all kinds of organs via tail vein injection in mice, compared with other formulations such as DDAB:Chol, DDAB:DOPE and so on. Secondly, DOTAP:Chol can provide more effective protection and delivery of DNA in circulation compared with other formulations, partly because of its more cohesive bilayer and its ability to effectively internalize DNA[23]. Lastly, intravenous injection of DOTAP:Chol-DNA complexes *in vivo *can induce significantly increased uptake of DOTAP:Chol-DNA complexes by tumor cells and angiogenic endothelial cells in tumors than by normal cells [31,32]. Moreover, previous studies showed that DOTAP:Chol-DNA complexes could target lung, which could protect lung from invasion of tumor cells [33,34]. All the evidences demonstrate cationic liposome (DOTAP:Chol) is an ideal gene delivery system in our study.

Before the in *vivo *test, we had made several studies concerning the effect of Msurvivin T34A plasmid on 4T1 cells *in vitro*. PI staining fluorescence microscopy and flow cytometric analysis of Msurvivin T34A plasmid treated tumor cells were conducted. The results of our findings were consistent with the apoptosis-inducing ability of Msurvivin T34A reported before [21]. These data suggest that Msurvivin T34A plasmid can induce the apoptosis of tumor cells directly.

*In vivo *test, we tested the Msurvivin T34A plasmid:lipo complexes on mouse metastatic breast cancer 4T1 model. We found that Msurvivin T34A plasmid efficiently inhibited the growth of 4T1 cancer. The treatment with Msurvivin T34A plasmid resulted in primary tumor growth regression of 73% and 57%, respectively, compared with NS and PORF-9 null plasmid groups(P < 0.05). From the results of examination of lungs, we found that the Msurvivin T34A plasmid treated mice had obvious reduction of tumor nodules on the lungs compared with control groups. To our delight, 4 of 10 mice had no visible lung metastases in the group treated with Msurvivin T34A plasmid. The results of H&E staining of lungs conformed to the visible observation. These results indicate that Msurvivin T34A plasmid has a potent ability to stimulate anti-tumor and anti-metastases activity in these mice with established 4T1 breast tumor.

Having witnessed the profound anti-tumor and anti-metastases activity of Msurvivin T34A plasmid *in vitro *and *in vivo*, we did TUNEL staining of each group to evaluate the anti-tumor mechanism *in vivo*. Our finding was also consistent with the apoptosis-inducing ability of Msurvivin T34A plasmid mentioned above [21].

We also observed that Msurvivin T34A plasmid could inhibit angiogenesis of tumor. Generation of blood vessels plays a crucial role in the growth and progression of most solid cancers[35,36]. It can influence the outcome of chemotherapy to inhibit angiogenesis[37]. Targeting tumor vasculature has been a new therapeutic strategy. It is reported that the tumor cells supported by the vessel would undergo apoptosis when endothelial cell apoptosis is induced [38]. In this study, Msurvivin T34A plasmid could induce obvious apoptosis of murine endothelial cells (MS1) *in vitro*, CD31 staining of Msurvivin T34A plasmid treated group showed the decrease of the number of intratumoral microvessel *in vivo *and alginate-encapsulated tumor cells test also confirmed that Msurvivin T34A plasmid could indeed affect angiogenesis. Furthermore, specific cytotoxic T cells can attack endothelial cell of tumor vessels, because the survivin is strongly expressed in tumor endothelial cell.

Previous studies also showed that lymphocytes in human tumor microenvironments are cytotoxic to the tumor cells by in situ observations of lymphocyte-tumor cell interaction[39,40]. Thus, active specific immunotherapies with cancer vaccine-based tumor antigens represent very promising approaches for cancer therapy[41,42]. The success of cancer immunotherapy is dependent on using suitable tumor-associated antigens. Survivin is an ideal tumor-associated antigen, for it is strongly expressed in most solid tumors but poorly expressed in adult normal tissues and it is obligatory for the survival of tumor cells. It is conceivable that tumors may not be able to escape an anti-survivin CTL response. Moreover, using Msurvivin T34A plasmid as a vaccine can eliminate the potentially serious problem of delivering a potent antiapoptotic activity associated with wild type survivin. In current study, we utilized Msurvivin T34A plasmid to produce specific cytotoxic T lymphocyte response, which can target to recognize and attack the tumor cells.

## Conclusion

Taken together, we demonstrate that the mouse survivin T34A plasmid complexed with cationic liposome can efficiently inhibit the growth and metastases of mouse highly metastatic mammary carcinoma *in vitro *and *in vivo*. The mechanism is involved in three aspects: triggering the apoptosis of tumor cells, inhibiting angiogenesis and inducing humoral and cellular immune response. Our findings collectively suggest that Msurvivin T34A plasmid can be considered as a new treatment approach for breast cancer.

## Competing interests

The authors declare that they have no competing interests.

## Authors' contributions

XCP carried out cell transfection, animal experiment, histologic analysis and drafted the manuscript. LY participated in the plasmid DNA preparation and animal experiment. LPY contributed to animal experiment and histologic analysis. YQM carried out flow cytometric analysis. HSY participated in its design. JYL performed the statistical analysis. DMZ helped to draft the manuscript. LJC carried out Liposome preparation. YQW supervised experimental work and revised the manuscript. All authors read and approved the final manuscript.
